# Camp Notes

**Published:** 1880-07

**Authors:** 


### CAMP NOTES.

Camp notes are better than bank notes—for health.

You can hear a little child's call, in the woods, farther than that of a tin horn.

There are no lovlier flowers than those that grow in the woods. I sometimes doubt whether florists have increased their beauty any by cultivation.

I am willing to wager a cookie that a little red ant of my acquaintance can whip any spider, caterpiller, or "thousand-legged worm" on the continent. Competition open till fall.

The wood-robin has not adhered strictly to his time for singing in camp. He is accredited with singing early in the morning and late at night. I have heard him at all hours of the day, and a very skilful musician he is, too.

There is some sort of a little warbler—no larger than a ground sparrow, that sings the same notes as the robin, but much sweeter. I have tried to get near the little chap, but have not succeeded in making his acquaintance. He commences his song about five o'clock in the evening, and keeps it up almost continually until sun-down.

A muskrat will swim gracefully about a clear pond, sit on a log and wash his hands and face, dine on a bunch of clover, and play and frolic within reach of your rod—as long as you are lying on the bank, motionless. But just stir a foot or wink your eye, and in a twinkling he is gone—and for the life of you, you can not tell where. A muskrat has the power of becoming invisible, not possessed by any other creature of my acquaintance.

A toad undresses to dress himself. He will scratch, and claw and squirm and blink and croak and tug at his old coat until he succeeds in pulling it off. Then, instead of sending it to the wash, he swallows it. We have seen clothing on some men that, if boiled, migh make nutritious soup, but we never supposed a garment would prove palatable raw. Evidently, the toad thinks differently. But how beautiful he appears in his new dress. His colors are quite pretty and his skin has the appearance of being varnished. He's a pretty creature when freshly dressed.

The King-fisher is a glutton. He eats continually. His hunger is never satisfied. He feeds his young on newts and swallows the trout himself.

The mosquito, like the rattlesnake, gives warning before he strikes, but the punkie is a contemptible copperhead, biting before declaring himself.

We have settled quite definitely this fact: when it commences raining before seven o'clock in the morning, it will quit before eleven. If a rain storm clears off at night, the heavens becoming clear, it will certainly rain next day. That is about the way it behaves in camp.

The depletion of the trout streams, it is suggested, is partly due to the enormous appetites of the visitors to camp, every season. A lady who would find it difficult to dispose of one trout at home, will speedily manage a half-dozen in camp, and will even then rise from the table with an expression of returning hunger upon her face. It would seem that out-door life is a great sharpener of the appetite.

"The patter, patter, patter of the rain upon the roof" is delightfully soothing and soon woos one into slumber. But, confound it, a fellow does not want to slumber *all* the while. Our slumbering inducements have been prolific, in camp, this year. When a bright day does come however, it is enjoyed the more thoroughly. It really matters little after all, whether it rains or shines, when you are out for a vacation. I could never perceive any difference in the amount or quality of rest obtained in storm or sunshine.

Folks imagine that water taken from a spring is pure and clean. I have never lain down to drink from a spring that I did not see on the bottom wiggling creatures of all sorts, and have occasionally taken them in when I did not close my suction soon enough—to the utter annihilation of the wiggler. Newts, rotifers, desmids, and a great variety of living creatures always abide in the springs, particularly in the winter, when the water is warm for them. The microscopist understands this fact and always goes to the springs to find living creatures for exhibition under the microscope. Do not therefore drink from a spring under the delusion that it is pure water—it is'nt!

